# Recipient Outcomes with Younger Donors Undergoing Living Donor Liver Transplantation

**DOI:** 10.7759/cureus.4174

**Published:** 2019-03-04

**Authors:** Faisal S Dar, Nusrat Y Khan, Rubab Ali, Hamzah Khawar Khokhar, Haseeb H Zia, Abu Bakar H Bhatti, Najmul H Shah

**Affiliations:** 1 Surgery, Shifa International Hospital, Islamabad, PAK; 2 Surgery, Shifa International Hospital, Islamabad , PAK

**Keywords:** liver transplantation, donors, mortality, allograft dysfunction

## Abstract

Introduction

The impact of donor age on liver transplantation is well known. Data on an appropriate donor age cut-off for living donor liver transplantation (LDLT) with a background of hepatitis C (HCV) is generally limited. The objective of this study was to determine whether limiting donor age to less than 35 years improved outcomes in patients with HCV-related end-stage liver disease (ESLD).

Methods

This was a retrospective review of 169 patients who underwent LDLT for HCV-related ESLD. The patients were divided into two groups based on whether they received grafts from donors ≤ 35 (Group 1) or > 35 (Group 2) years of age. Kaplan Meier curves were used to determine survival. Uni and multivariate analysis were performed to determine independent predictors of mortality.

Results

Mean donor age was 25.1 ± 5.2 and 40.1 ± 3.4 years (P < 0.0001). Early allograft dysfunction (EAD) was seen in 11.7% patients in Group 1 versus 29.6% in Group 2 (P = 0.02). A significant difference in mortality was present between the two groups, i.e., 33.3% versus 15.8% (P = 0.04). The estimated four-year overall survival (OS) was 78% and 64% (P = 0.03). Upon doing univariate analysis, the donor age (P = 0.04) and EAD (P = 0.006) were found to be significant variables for mortality. On multivariate analysis, EAD was the only independent predictor of mortality (Hazard ratio: 2.6; confidence interval: 1.1 - 5.8; P = 0.01).

Conclusion

Opting for younger donors (≤ 35 years) for HCV-related ESLD patients lowers the risk of EAD and improves overall survival.

## Introduction

Living donor liver transplantation (LDLT) is an invaluable therapeutic modality in regions with deceased donor organ shortage [1]. Several donor and recipient factors impact outcomes after transplantation and donor age is one of them [2-3]. Donor risk index (DRI) is a widely used criterion that utilizes various donor characteristics to stratify the risk of post-transplant graft loss [4]. Other than donor age and height, none of the DRI variables are applicable to the LDLT setting. The literature on the appropriate donor age cut-off for LDLT with background hepatitis C virus (HCV) is generally limited [5]. Moreover, in the presence of HCV, which has been linked with a higher rate of graft failure and mortality, the evidence is lacking regarding an appropriate age cut-off [6]. After age 30, there is a progressive loss of liver volume and blood flow. These changes lead to the progressive deterioration of the liver’s response to situations with high metabolic demands [7-9]. It has been shown that in adult LDLT, a mean donor age of 34.4 years is protective against early graft dysfunction versus an age of 43.1 years [10].

We have been very cautious in our donor selection in terms of donor age compared to other programs [11]. The age cut-off used at our center is 18-45 years. Despite this age cut-off, we have experienced biliary complications, HCV recurrence, early allograft dysfunction (EAD), and mortality in some patients. Would a younger donor age cut off (≤ 35 years) confer any outcome benefit for patients undergoing LDLT for HCV-related end-stage liver disease (ESLD)? The objective of this study was to determine if donor age < 35 years improves outcomes in transplanted patients with a HCV-positive ESLD.

## Materials and methods

This was a retrospective review of patients who underwent LDLT. Until April 2016, 303 LDLTs were performed at the Shifa International Hospital, Islamabad. A total of 169 patients who underwent LDLT for HCV-related ESLD were included in the study. Patients with acute liver failure, age less than 18 years, dual graft, domino recipient, and those transplanted after 30th October 2015 were excluded.

The details of the donor and recipient evaluations and selection have been reported elsewhere [1, 12]. Donors were 18-45 years of age, blood group compatible, and related to recipients. The decision to transplant was made after receiving approval from the Human Organ and Tissue Transplant Authority (HOTA) of Pakistan and a transplant listing meeting.

The donor/recipient characteristics and operative variables were assessed. For this study, the patients were divided into two groups based on donor age; Group 1 had grafts from donors aged 35 years and below and Group 2 had donors aged 36 years and above. Donor characteristics were compared between the two groups including gender, body mass index (BMI), and graft and operative variables. A comparison was also made for recipient characteristics including age, gender, BMI, child Turcot Pugh score (CTP) score, model for end-stage liver disease (MELD) score, the presence of hepatocellular carcinoma (HCC), and operative variables. In terms of outcomes, the following variables were assessed; 1) early allograft dysfunction (EAD); 2) HCV recurrence; 3) biliary complications; 4) 90-day mortality; 5) overall mortality. Biliary complications were categorized based upon Clavien Dindo grading [13]. EAD was defined as the presence of one or more of the following: Bilirubin ≥10 mg/dl on Day 7, international normalized ratio (INR) ≥ 1.6 on Day 7, and alanine or aspartate aminotransferase > 2,000 IU/L within the first seven days after liver transplantation [14]. HCV recurrence was defined as a positive polymerase chain reaction (PCR) in the presence of rising liver function tests (LFTs).

For categorical variables, the Chi-Square test and the Fisher exact test were used, while for interval variables, independent tests were applied. For survival, Kaplan Meier curves were generated, and log rank tests were used to determine significance. Overall survival was calculated by subtracting date of death/last follow up from date of transplant. A Cox proportional hazard model was used to determine independent predictors of patient survival. Variables with a P-value < 0.1 were considered for univariate analysis and significant variables (P < 0.05) on univariate analysis were included in the multivariate analysis. All analysis was performed on Statistical Package for Social Sciences (SPSS) version 20 (IBM, Armonk, New York, United States). The study was approved by the hospital ethics committee.

## Results

Donor and recipient characteristics

Table 1 demonstrates various donor and recipient variables. Majority of donors in Group 1 were males, i.e., 109 (75.1%) versus 12 (50%) (P = 0.01). Mean donor age was 25.1 ± 5.2 and 40.1 ± 3.4 years (P < 0.0001). Mean donor BMI was 23.9 ± 3.8 and 27.3 ± 3.2Kg/m2 for the two groups (P < 0.0001). Mean LAI was 11 ± 5.9 and 9.8 ± 5.3 (P = 0.3). Mean recipient age was 48.6 ± 8.1 and 45.6 ± 7.6 years (P = 0.1). Mean recipient BMI was 24 ± 3.7 and 23.9 ± 3.8Kg/M2 (P = 0.7). Mean MELD score was 17.2 ± 6.1 and 16.9 ± 7.9 (P = 0.7). Mean cold ischemia time was 47.4 ± 29.9 and 37 ± 21.4 minutes (P=0.1). 

**Table 1:** Donor and recipient characteristics of the study cohort

Donor characteristics		Group 1 N=145		Group 2 N=24		
		Number	Percent	Number	Percent	P value
Gender	Male	109	75.1	12	50	0.01*
	Female	36	24.9	12	50	
Lobe	Right	142	97.9	23	95.8	0.4
	Left	3	2.1	1	4.2	
Middle hepatic vein used	Yes	34	23.4	2	8.3	0.09
Arterial anatomy	Standard	92	63.4	11	45.8	0.1
Biliary anatomy	Standard	56	38.6	9	37.5	0.9
Recipient characteristics						
Gender	Male	119	82	21	87.5	0.3
	Female	26	18	3	12.5	
Child Turcot Pugh grade	A	7	4.8	2	8.3	0.6
	B	59	40.7	8	33.4	
	C	79	54.5	14	58.3	
Hepatocellular carcinoma	Present	37	25.5	7	29.1	0.8

Comparison of outcomes

No significant differences were observed between the two groups in terms of biliary complications and HCV recurrence. There was a statistically significant difference in EAD and overall mortality as shown in Table 2. EAD was seen in 11.7% of the patients in Group 1 versus 29.6% in Group 2 (P = 0.02). Overall, the mortality was significantly high in Group 2 patients, i.e., 33.3% versus 15.8% (P = 0.04). Since there was a significant difference in the gender distribution between the two groups as shown in Table 1, the impact of gender on EAD and mortality was separately assessed in Group 1 patients. Overall mortality for males and females was 17.4% (19/109) versus 11.1% (4/36) and was not significantly different (P = 0.4).

**Table 2:** Comparison of outcomes between the two groups

	Group 1 N=145		Group 2 N=24		
	Number	Percent	Number	Percent	P value
Hepatitis C virus recurrence	54	37.2	9	37.5	0.9
Early allograft dysfunction	17	11.7	7	29.1	0.02*
Biliary complications	30	20.6	6	25	0.6
90 day mortality	13	8.9	5	20.8	0.08
Overall Mortality	23	15.8	8	33.3	0.04*

Predictors of survival

**Figure 1:**
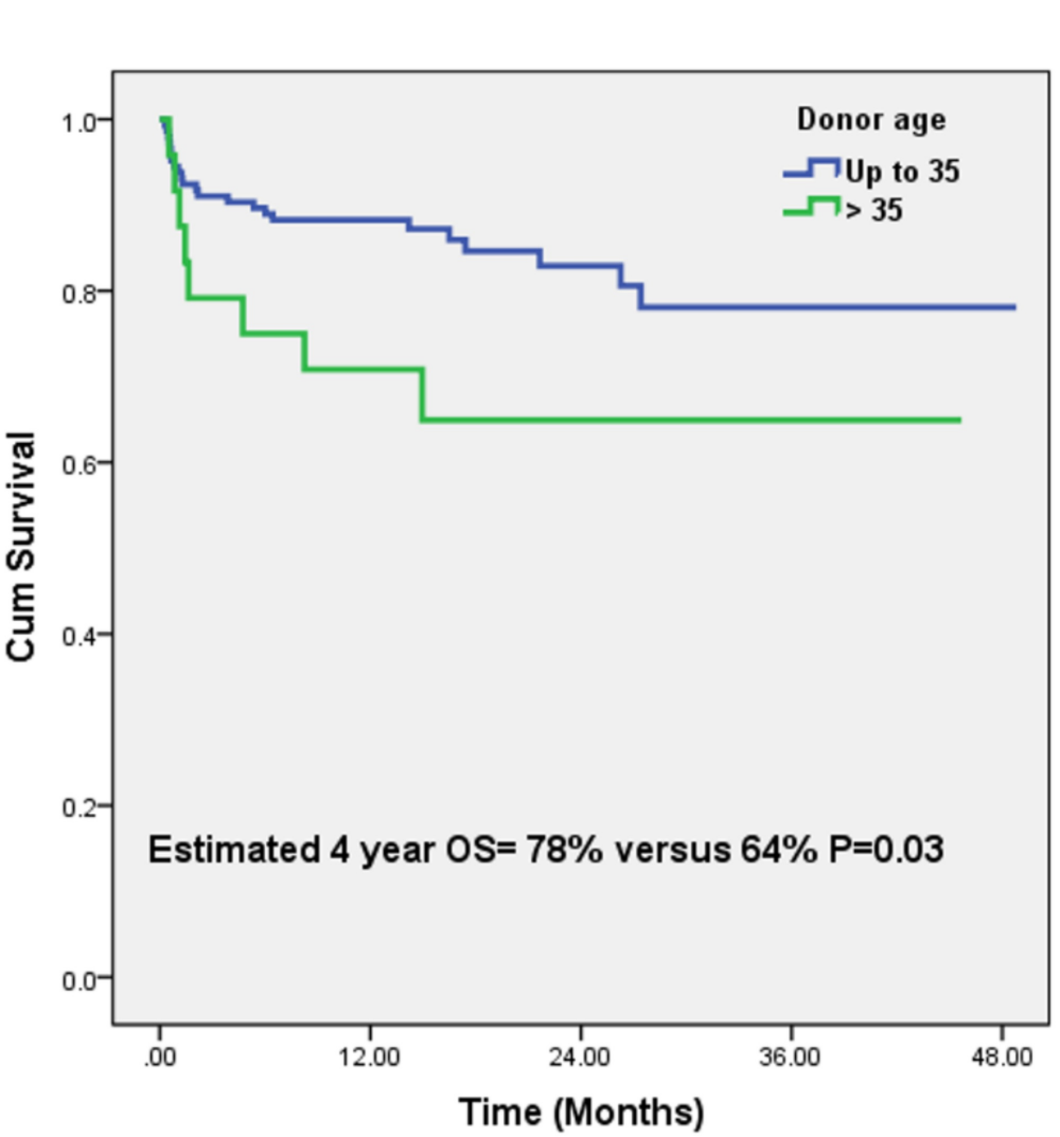
Estimated four-year overall survival in patients with HCV related end-stage liver disease with donor age cut-off of 35 years HCV: hepatitis C virus

Estimated one-year overall survival (OS) for the whole group was 87%. Estimated one-year OS for Groups 1 and 2 were 88% and 71% and estimated four-years OS were 78% and 64% and was significantly different (P = 0.03) (Figure 1). We included gender, BMI, and MHV use in our univariate analysis for mortality as shown in Table 3. On multivariate analysis, EAD was the only independent predictor of survival and resulted in a significant increase in the risk of death [HR: 2.6; CI: 1.1 - 5.8; P = 0.01]. There was a major reduction in risk of death post-transplantation in the group with donors <35 years age secondary to lower rates of EAD.

**Table 3:** Univariate and multivariate analysis for post transplant mortality

	Variables		Hazard ratio	95% confidence interval	P value
Univariate analysis					
Mortality	1) Male gender	1.4		0.6-3.2	0.4
	Female	1			
	2) Body mass index ≤ 25	1		0.9-1.1	0.8
	>25	1			
	3) Middle hepatic vein used	1.03		0.6-1.6	0.8
	Not used	1			
	4) Early allograft dysfunction present	0.33		0.1-0.7	0.006*
	Absent	1			
	5) Donor age ≤35	0.4		0.1-0.9	0.04*
	>35	1			
Multivariate analysis					
Mortality	1) Early allograft dysfunction present	2.6		1.1-5.8	0.01*
	absent	1			
	2) Donor age ≤ 35	0.5		0.2-1.1	0.1
	Age >35	1			

## Discussion

The current study demonstrates the outcomes of LDLT recipients for HCV-related ESLD. Donors were younger with a male predilection and the majority of patients were CTP grade C at the time of transplantation. Younger donor age (≤ 35 years) conferred survival advantage secondary to the lower rates of EAD. Variable donor age cut-offs have been used in different studies. This represents differences in the unique genetic makeup of the individual population, etiology of liver failure, and DDLT versus LDLT [15-18]. A number of studies have addressed donor ages in LDLT [16-21]. The outcomes remain variable with some studies demonstrating an inferior recipient survival and increased frequency of small for size syndrome, while others show no impact on outcomes. Han and colleagues compared recipient outcomes in 604 LDLTs and found that a donor age cut-off of 55 years to be associated with increased recipient mortality [11]. On the other hand, Li and colleagues in their study on 129 LDLTs demonstrated acceptable and comparable survival with donors greater than or lesser than 70 years of age [18]. Ikegami and colleagues demonstrated a higher rate of small for size syndrome with donors older than 50 years [19]. There is no literature on LDLT that solely demonstrates the impact of having a younger donor age (≤ 35 years) in HCV-positive recipients. In the current study, 169 HCV-related ESLD recipients were included, and a relatively younger donor age compared to other studies was found to significantly impact the EAD and, thus, mortality.

Several prognostic factors have been proposed to identify the risk factors for graft loss after transplantation [4]. Important factors like cold ischemia time (CIT), cause of death, allocation system, and race are not relevant to LDLT since the CIT time is short in LDLT, all donors are alive, and allocation is not required since patients do not compete for organs in the presence of individualized donations. Other factors including recipient age, BMI, CTP, and MELD score were evenly distributed in the two groups.

The current study demonstrates superior survival with donors ≤ 35 years in a relatively younger donor cohort (maximum age 45 years). The rate of biliary complications in our cohort was similar between the two groups probably because biliary complications have been associated with relatively older and extended criteria donors [22]. Donor age has been associated with EAD, and it was the only significant variable on univariate analysis in the current study [23]. No significant difference in HCV recurrence was observed in the current study. A majority of the deaths occurred within the first 90 days after transplantation and were attributable to sepsis which itself can be a sequel of EAD [23]. HCV genotype 3 is the predominant genotype in the subcontinent. It has been associated with an increased rate of fibrosis and a higher incidence of HCC [24]. Furthermore, the response to direct acting anti-viral (DAA) medications is still not well understood, but genotype 3 has been associated with a lower response rate than other genotypes [25]. A relatively short follow-up and frequent use of DAAs might have a plausible role in the current study with very few mortalities attributable to HCV recurrence. 

Without much impact on other outcome variables, the younger donor age had a significant impact on the overall survival of transplanted patients. Donor gender and BMI were the only variables that were unevenly distributed between the two groups but did not impact survival on univariate analysis. In order to confirm the impact of gender, we also looked for the gender-specific rate of EAD and mortality in recipients of grafts ≤35 years of age and found no significant difference. Several mechanisms have been proposed to explain changes in liver architecture with increasing age. Perhaps the most widely understood is telomere shortening, which is most active in the 30s [26-27]. Similarly, it has been shown that a mean age of 34.4 years might be protective for EAD for reasons yet unknown. The limitations of the current study include its retrospective design and the smaller number of younger donors. However, considering that donors < 35 years of age represent a very select group of donors, it still represents a significant number.

The current study identified EAD as an independent predictor of mortality and was seen more frequently in an older donor age (>35 years). The Survival for patients who received grafts from younger donors was 88% versus 71% at one year. Clearly, one-year survival of 71% is below the international average, but an overall one-year survival of 87% for HCV-related ESLD is very much in line with international recommendations [28].

## Conclusions

The current study demonstrates a major reduction in the risk of death post-transplantation in the group with donors < 35 years age secondary to lower rates of EAD. The ideal donor age cut-off remains debatable due to the interplay of multiple donor, recipient, and treatment-related variables that impact graft dysfunction. It is unclear if larger studies with longer follow-up can better answer this question, but they can definitely enhance our understanding of this important variable and how it relates to patient outcomes.
